# Anemia and Iron Deficiency in Outpatients with Inflammatory Bowel Disease: Ubiquitous Yet Suboptimally Managed

**DOI:** 10.3390/jcm11226843

**Published:** 2022-11-19

**Authors:** Roberta Loveikyte, Menno Boer, Catharina N. van der Meulen, Rinze W. F. ter Steege, Greetje Tack, Johan Kuyvenhoven, Bindia Jharap, My K. Vu, Lauran Vogelaar, Rachel L. West, Sander van der Marel, Tessa E. H. Römkens, Zlatan Mujagic, Frank Hoentjen, Adriaan A. van Bodegraven, Fiona D. M. van Schaik, Annemarie C. de Vries, Gerard Dijkstra, Andrea E. van der Meulen-de Jong

**Affiliations:** 1 Department of Gastroenterology and Hepatology, Leiden University Medical Center, Leiden University, 2333 ZA Leiden, The Netherlands; 2 Department of Gastroenterology and Hepatology, University Medical Center Groningen, University of Groningen, 9713 GZ Groningen, The Netherlands; 3 Department of Gastroenterology and Hepatology, Martini Hospital, 9728 NT Groningen, The Netherlands; 4 Department of Gastroenterology and Hepatology, Medical Center Leeuwarden, 8934 AD Leeuwarden, The Netherlands; 5 Department of Gastroenterology and Hepatology, Spaarne Gasthuis Hospital, 2000 AK Haarlem, The Netherlands; 6 Department of Gastroenterology and Hepatology, Meander Medical Center, 3813 TZ Amersfoort, The Netherlands; 7 Department of Gastroenterology and Hepatology, Alrijne Hospital, 2350 CC Leiderdorp, The Netherlands; 8 Department of Gastroenterology and Hepatology, Diakonessenhuis Hospital, 3582 KE Utrecht, The Netherlands; 9 Department of Gastroenterology and Hepatology, Franciscus Gasthuis & Vlietland Hospital, 3004 BA Rotterdam, The Netherlands; 10 Department of Gastroenterology and Hepatology, Haaglanden Medical Center, 2512 VA The Hague, The Netherlands; 11 Department of Gastroenterology and Hepatology, Jeroen Bosch Hospital, 5223 GZ Den Bosch, The Netherlands; 12 Department of Gastroenterology and Hepatology, Maastricht University Medical Center+, Maastricht University, 6229 HX Maastricht, The Netherlands; 13 Department of Gastroenterology and Hepatology, Radboud University Medical Center, Radboud University, 6525 GA Nijmegen, The Netherlands; 14 Division of Gastroenterology, University of Alberta, Edmonton, AB T6G 2X8, Canada; 15 Department of Gastroenterology, Geriatrics, Internal- and Intensive Care Medicine (COMIK), Zuyderland Medical Center, 6130 MB Sittard-Geleen, The Netherlands; 16 Department of Gastroenterology and Hepatology, University Medical Center Utrecht, Utrecht University, 3584 CX Utrecht, The Netherlands; 17 Department of Gastroenterology and Hepatology, Erasmus University Medical Center, Erasmus University Rotterdam, 3015 GD Rotterdam, The Netherlands

**Keywords:** anemia, iron deficiency, Inflammatory Bowel Disease

## Abstract

Background: Iron deficiency (ID) and anemia in patients with Inflammatory Bowel Disease (IBD) are associated with a reduced quality of life. We assessed the prevalence of ID and anemia in Dutch outpatients with IBD and compared routine ID(A) management among medical professionals to the European Crohn’s and Colitis Organisation (ECCO) treatment guidelines. Methods: Between January and November 2021, consecutive adult outpatients with IBD were included in this study across 16 Dutch hospitals. Clinical and biochemical data were extracted from medical records. Additionally, medical professionals filled out questionnaires regarding routine ID(A) management. Results: In total, 2197 patients (1271 Crohn’s Disease, 849 Ulcerative Colitis, and 77 IBD-unclassified) were included. Iron parameters were available in 59.3% of cases. The overall prevalence of anemia, ID, and IDA was: 18.0%, 43.4%, and 12.2%, respectively. The prevalence of all three conditions did not differ between IBD subtypes. ID(A) was observed more frequently in patients with biochemically active IBD than in quiescent IBD (ID: 70.8% versus 23.9%; *p* < 0.001). Contrary to the guidelines, most respondents prescribed standard doses of intravenous or oral iron regardless of biochemical parameters or inflammation. Lastly, 25% of respondents reported not treating non-anemic ID. Conclusions: One in five patients with IBD suffers from anemia that—despite inconsistently measured iron parameters—is primarily caused by ID. Most medical professionals treat IDA with oral iron or standard doses of intravenous iron regardless of biochemical inflammation; however, non-anemic ID is often overlooked. Raising awareness about the management of ID(A) is needed to optimize and personalize routine care.

## 1. Introduction

Anemia is the most common systemic manifestation of Inflammatory Bowel Disease (IBD): Crohn’s disease (CD), Ulcerative Colitis (UC), and IBD-unclassified (IBD-U). The reported prevalence varies between 6% and 74% [1,2]. Patients with IBD are susceptible to anemia due to chronic gastrointestinal blood loss, medication-induced myelosuppression, and nutritional deficiencies caused by malnutrition or micronutrient malabsorption. Despite the plethora of predisposing factors, the most common causes of anemia in IBD are absolute iron deficiency (ID) and functional ID, which is characterized by inflammation-induced myelosuppression and iron restriction within enterocytes, hepatocytes, and macrophages. The reported prevalence of ID in IBD varies between 36% and 90% [1]. Data on the prevalence of ID and anemia, including risk factors, are inconsistent due to heterogeneous study populations and ID(A) definitions.

Iron is essential for most physiological processes such as energy metabolism, oxygen transport, immune system function, and neurotransmitter synthesis [3]. Consequently, ID can cause various symptoms ranging from dry skin to depression or a significant decrease in physical and cognitive performance [4,5]. Anemia is associated with a reduced quality of life (QoL), worse disease outcomes, and increased healthcare costs in patients with IBD [5,6,7]. Non-anemic ID is also associated with fatigue and decreased health-related QoL that may be improved with iron therapy [6,7,8,9,10]. Therefore, the latest European Crohn’s and Colitis Organisation (ECCO) guidelines emphasize the importance of regular screening and prompt treatment of (iron-deficiency) anemia in patients with IBD [11].

Diagnosis of absolute and functional ID is challenging in patients with IBD [12,13,14]. Iron indices, such as ferritin or transferrin, can be difficult to interpret due to their dual role as acute-phase reactants, i.e., ferritin increases and transferrin decreases during (systemic) inflammation that could falsely indicate sufficient iron stores [15]. In addition, patients with IBD often suffer from inflammation that can restrict enteral iron absorption and lead to excess luminal iron that—according to data from animal studies—might exacerbate the disease [16]. Despite a lack of consistent research on hepcidin (the systemic iron regulator), iron absorption, and the effect of excess luminal iron in patients with IBD, the latest ECCO guidelines suggest intravenous iron as the first-line treatment in active disease and oral iron only in quiescent or mild disease [11]. ECCO guidelines were published in 2015, but their clinical implementation has not yet been evaluated.

In this study, we evaluated the prevalence of anemia and ID in patients with IBD under ambulatory care across 16 Dutch hospitals. In addition, we assessed routine ID(A) management among Dutch (trainee) gastroenterologists and IBD nurses. This study addresses aspects of routine care that need optimization.

## 2. Materials and Methods

### 2.1. Study Design and Study Population

Between January and November 2021, we conducted a national cross-sectional study across 16 general, teaching, and academic hospitals within the Netherlands. The study has been reviewed and approved by the Medical Ethics Review Board at the University Medical Center Groningen (METc No. 2020/549) and local committees or review boards at the participating centers. All consecutive outpatients with IBD were screened for inclusion during the study period. Patients were eligible for inclusion if they were at least 18 years old, had an established IBD diagnosis, and had a recent follow-up appointment with available biochemical data. Hospitalized patients and patients with the following documented comorbidities were excluded: portal hypertension, myelodysplastic syndrome, hemoglobinopathies, autoimmune hemolytic anemia, end-stage renal disease (defined as estimated Glomerular Filtration Rate <30 mL/min/1.73^2^), and a history of malignancy within the last five years except for cutaneous (non-melanoma) malignancies. Pregnant or breastfeeding women were also excluded. Each participating hospital included consecutive patients that met the in- and exclusion criteria with the aim to include 100 patients with sufficient data on iron status but without exceeding 200 inclusions per hospital.

### 2.2. Data Collection

Demographic and clinical data (detailed in Table 1) and the following biochemical data were extracted from medical records: hemoglobin (Hb), hematocrit (Ht), Mean Corpuscular Volume (MCV), white blood cell count (WBC), platelets, C-reactive protein (CRP), lactate dehydrogenase (LDH), reticulocytes, ferritin, serum iron, transferrin, total iron-binding capacity (TIBC), transferrin saturation (Tsat), vitamin B12 and folic acid levels, as well as fecal calprotectin (FCP). Clinical and biochemical data were extracted at inclusion: biochemical data could not be older than eight weeks. In addition, biochemical data were extracted retrospectively from a previous follow-up appointment, which depending on the disease activity and follow-up frequency, varied from three to 12 months prior to inclusion. If a patient was newly referred to the hospital, the data were extracted only at inclusion. Finally, clinical data on iron therapy (i.e., dose and the route of administration), including any phosphate measurements between the two follow-up appointments, were also extracted from medical records.

### 2.3. Study Definitions

CD was classified according to the Montreal classification: age at diagnosis (Montreal A), disease location (Montreal L), perianal disease (Montreal p), and disease behavior (Montreal B) [17,18]. UC and IBD-U were classified according to age at diagnosis (Montreal A) and disease extension (Montreal E) [17,18]. Montreal classification was extracted from medical records.

Anemia definition was based on the Dutch national reference values: Hb < 7.5 mmol/L (<120.7 g/L) or <8.5 mmol/L (<137.0 g/L) for females and males, respectively [19]. Severe anemia was defined as Hb <6.2 mmol/L for females and males. ID was defined as ferritin <100 µg/L in patients with biochemical inflammation (defined as CRP > 5 mg/L and/or FCP >150 mg/kg), or as ferritin <30 µg/L in patients without biochemical inflammation [11]. If ferritin values were unavailable, Tsat <20% was used regardless of biochemical inflammation. Tsat values were extracted from medical records or were calculated as: (serum iron concentration (µmol/L)/TIBC (µmol/L)) × 100 or (serum iron concentration (µg/dL)/transferrin (mg/L)) × 71.24. Moreover, IDA was defined as concurrent anemia and ID; functional iron deficiency (FID) was defined as Tsat <20% in combination with ferritin > 100 µg/L in states of biochemical inflammation [11]. Lastly, vitamin B12 concentration < 150 pmol/L and folic acid concentration < 5 nmol/L were defined as deficiencies [20,21].

### 2.4. Questionnaire for Medical Professionals

An anonymous questionnaire was drafted in Dutch and was sent to all medical professionals treating patients with IBD at the Department of Gastroenterology and Hepatology in the participating hospitals. It included questions regarding respondents’ medical profession (e.g., nurse practitioner, gastroenterologist trainee), workplace (e.g., academic hospital), the number of weekly IBD outpatient consultations, and the type/frequency of ID(A) screening in patients with active or quiescent IBD. In addition, questionnaires included questions regarding the treatment of asymptomatic or symptomatic anemia and non-anemic ID. The respondents were asked to indicate prescribed oral and intravenous iron dosages and whether they prescribed a standard dosage or based it on a specific formula. Lastly, respondents were asked whether they measured phosphate levels before and/or after intravenous iron therapy.

### 2.5. Statistical Analysis

Data extracted at inclusion were used to analyze the prevalence of anemia, ID, and IDA. Descriptive data are reported as mean values with Standard Deviation (±SD) or median values with an interquartile range [IQR: Quartile 1–Quartile 3] for continuous variables. Categorical variables are presented as a number (*n*) of the total available data with corresponding percentages (%). Normality assessment was performed by visual inspection of normal probability plots (Q-Q) and histograms. Prevalence of anemia and ID are presented as absolute numbers (*n*) with corresponding percentages (%). Group comparisons were analyzed using chi-square, Mann-Whitney U, unpaired t-test, or Kruskal Wallis-test as appropriate.

In addition, univariable and multivariable binary logistic regression analyses were performed to identify parameters independently associated with the prevalence of anemia, ID, or IDA. Multivariable analyses were performed using backward stepwise selection (*P_out_* > 0.05), with the inclusion of all significantly (*p* < 0.05) associated variables from the univariable analyses. Furthermore, we used data from the previous follow-up appointment and the anonymous questionnaire to analyze the frequency of ID(A) treatment and the type of iron therapy. The data are presented as a number (*n*) of the available data with corresponding percentages (%). A two-tailed *p*-value < 0.05 was considered statistically significant. The Benjamini-Hochberg procedure was used to adjust for multiple testing with a 5% false discovery rate. Statistical analyses were performed using SPSS software (version 25.0; IBM, Chicago, IL, USA). GraphPad Prism software (version 9.3.1; GraphPad Software, San Diego, CA, USA) was used to generate donut charts. Missing data are presented in Supplementary Tables S1 and S2.

## 3. Results

### 3.1. Study Population

In total, 2197 patients with IBD (CD: *n* = 1271, UC: *n* = 849, IBD-U: *n* = 77) were included in the study. Patients were analyzed by stratifying them into CD and UC groups, the latter included patients with UC and IBD-U. Table 1 presents the baseline demographic and clinical characteristics of the study population. Patients with CD were primarily female (61.4%) and significantly younger compared with patients in the UC group (*p* < 0.001). Biochemical inflammation was more frequently observed in patients with CD than in patients with UC (*p* < 0.01).

### 3.2. Prevalence of Anemia, Iron Deficiency, and Iron-Deficiency Anemia

The data on the prevalence of anemia, ID, and IDA in the study population are presented in Table 1. In general, 18% of patients with IBD had anemia. Only 28 patients (7.1% of patients with anemia) had severe anemia. There was no difference in anemia prevalence between patients with CD and UC, but anemia was more prevalent in males than females (22.8% vs. 14.5% for males and females, respectively; *p* < 0.001).

The etiology of anemia could not be classified in all patients: 59.3% of patients had sufficient data on iron status and biochemical inflammation. ID was prevalent in 43.4% of the study population without differences between patients with CD or UC (Table 1). On the other hand, ID prevalence differed between males and females (31.5% vs. 51.8%; *p* < 0.001), as well as patients with and without biochemical inflammation: 70.8% versus 23.9% in biochemically active or quiescent IBD, respectively (*p* < 0.001). In contrast, there were no differences in IDA prevalence between patients with CD and UC or males and females (Table 1).

### 3.3. Differences between Iron-Deficient and Iron-Sufficient Patients

Table 2 presents the differences between patients with and without ID. Median Hb, Ht, and MCV levels were lower in the iron-deficient group (*p* < 0.001 for all). In addition, inflammatory parameters—FCP, CRP, WBC, and platelets—were higher in patients with ID (*p* < 0.001 for all). No differences were observed in LDH, vitamin B12, or folic acid levels. Similar findings were observed between patients with and without anemia (Supplementary Table S4). In short, patients with ID have significantly lower Hb and higher inflammatory parameters compared with iron-sufficient patients.

### 3.4. Risk Factors for Anemia and Iron Deficiency

Male gender and older age were independently associated with anemia, as depicted in Table 3. In multivariable analyses, a two-fold increase in FCP [OR 1.18; 95% CI: 1.08–1.28; *p* < 0.001] and platelets [OR 2.17; 95% CI: 1.25–3.79; *p* < 0.01] as well as lower ferritin and MCV were associated with anemia. ID and IDA were also associated with a two-fold increase in FCP and platelet levels, but in contrast to anemia, younger age and female gender were associated with ID. Females were at least two times as likely to have ID compared with males [OR 2.63; 95% CI: 1.84–3.75; *p* < 0.001]; however, IDA was not related to age or gender (Table 3). In addition, the Montreal classification showed several statistically significant results in univariable analyses, but the significance was not observed in multivariable analyses (Supplementary Table S5). Stricturing disease (Montreal B2) was associated with a higher anemia prevalence compared with inflammatory disease phenotype (Montreal B1): OR 1.55 [1.12–2.16]; *p* < 0.01 (Supplementary Table S5). In short, biochemical inflammation is a major risk factor for anemia and ID(A) in all patients with IBD. However, older males are more likely to have anemia, while younger females are more likely to have ID.

### 3.5. Iron Therapy in the Study Population

**Figure 1:**
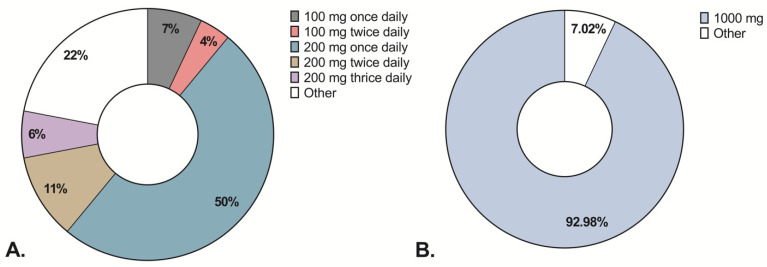
Prescribed oral and intravenous iron dose in patients with Inflammatory Bowel Disease. (**A**) Depicts the prescribed oral iron dosage. (**B**) Depicts prescribed intravenous iron dosage. The represented data are based on patient data extracted from medical records.

Compared to data at inclusion, iron parameters were measured less frequently at a previous appointment (in 47% of patients compared with 59.3% of patients at inclusion); the prevalence of all conditions was similar: ID 43.6%, IDA 6.9%, and anemia 18.6%. In total, 38.5% of patients with ID and 51.9% with anemia had these conditions at inclusion and a previous appointment regardless of iron therapy. Based on extracted retrospective data, 204 patients received iron therapy within the last 12 months. The majority of patients (55.9%) received intravenous iron, but only 5.8% of patients had their phosphate levels measured after the treatment (Table 4). Almost all patients received 1000 mg of intravenous iron, whereas oral iron dosage varied considerably (Figure 1).

Table 4 presents the differences between patients who received iron therapy and those who did not. Biochemical inflammation was more common in patients treated with intravenous iron than oral iron (67.0% vs. 48.8%, respectively). Most treated patients had IDA, but 34.5% (n = 37) of treated patients had anemia of a different etiology (e.g., FID) or anemia that could not be classified due to missing data, i.e., iron indices or vitamin B12 levels. In contrast, 36.7% of patients with ID did not receive any iron therapy (Table 4).

### 3.6. Iron Deficiency and Anemia Management: Responses from Medical Professionals

**Figure 2:**
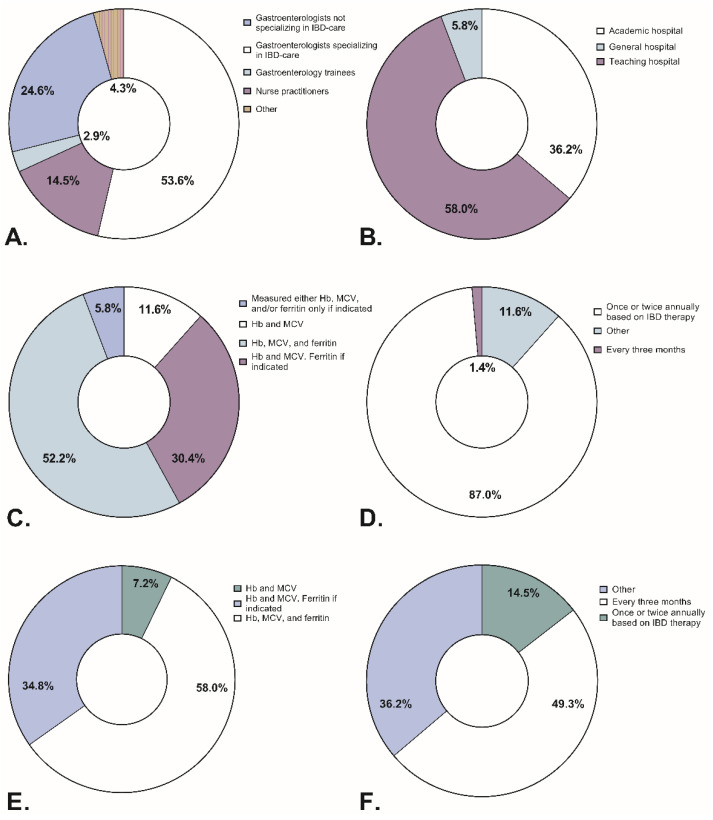
Respondent data regarding profession, place of employment, and screening for anemia and iron deficiency in patients with Inflammatory Bowel Disease. (**A**) Percentages indicating respondents’ profession. (**B**) Percentages indicating respondents’ place of employment. (**C**) Percentage of respondents and the parameters used to screen for anemia and iron deficiency in patients with quiescent Inflammatory Bowel Disease. (**D**) Percentage of respondents and the frequency of screening for anemia and iron deficiency in patients with quiescent Inflammatory Bowel Disease. (**E**) Percentage of respondents and the parameters used to screen for anemia and iron deficiency in patients with active Inflammatory Bowel Disease. (**F**) Percentage of respondents and the frequency of screening foranemia and iron deficiency in patients with active Inflammatory Bowel Disease. Hb: hemoglobin, MCV: Mean Corpuscular Volume.

The anonymous questionnaire was sent out to 112 medical professionals across 16 participating hospitals: 69 (61.6%) respondents filled out the anonymous questionnaire. Respondents’ professions and places of employment are represented in Figure 2. At least half of the respondents reported using Hb, MCV, and ferritin to screen for anemia and iron deficiency in patients with active or quiescent IBD, but a third of respondents reported measuring ferritin only on indication (Figure 2). In patients with quiescent IBD, almost all respondents (87%) reported screening once or twice annually, whereas in active disease screening is done more frequently (Figure 2).

**Figure 3:**
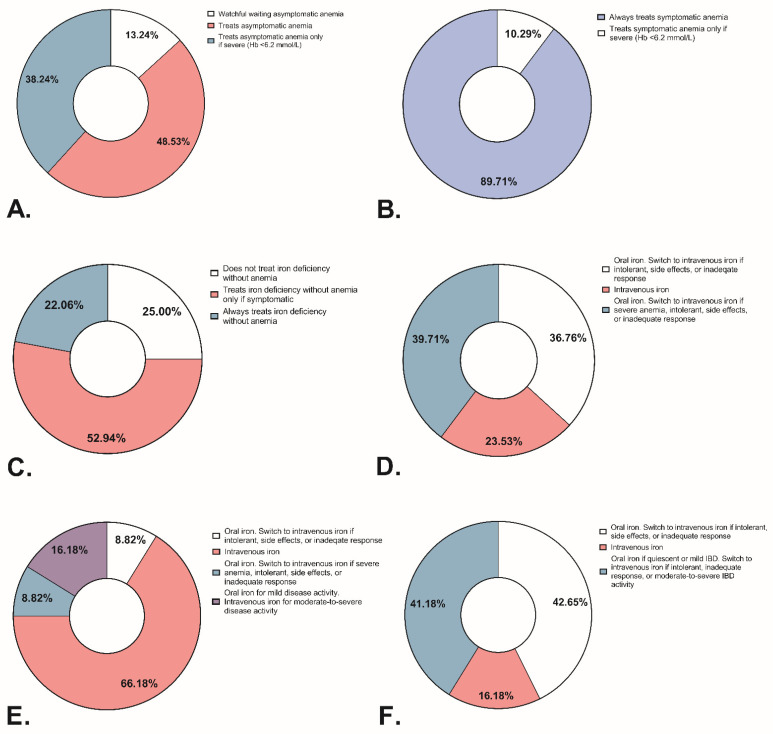
Respondent data regarding the treatment of iron deficiency with or without anemia in patients with Inflammatory Bowel Disease. (**A**) Percentage of respondents prescribing treatment for asymptomatic anemia. (**B**) Percentage of respondents prescribing treatment for symptomatic anemia. (**C**) Percentage of respondents prescribing treatment for iron deficiency without anemia. (**D**) Percentage of respondents and their chosen therapy for iron-deficiency anemia in quiescent IBD. (**E**) Percentage of respondents and their chosen therapy for iron-deficiency anemia in active IBD. (**F**) Percentage of respondents and their chosen therapy for iron deficiency without anemia. Hb: hemoglobin.

Figure 3 presents responses regarding the management of (a)symptomatic anemia and non-anemic ID. Comparing treatment policies between physicians and nurse practitioners, we observed only one statistically significant difference: 96.4% of physicians reported always treating symptomatic anemia, whereas 38.5% of nurse practitioners reported treating symptomatic anemia only if it is severe. Comparing treatment policies between academic and non-academic hospitals, we observed no significant differences.

In cases of non-anemic ID, 22% of respondents reported prescribing treatment regardless of symptoms, while 25% of respondents did not treat it at all. In contrast to non-anemic ID, most respondents (89.7%) reported treating symptomatic anemia regardless of severity. Oral iron was the preferred treatment in quiescent IBD, whereas intravenous iron in active IBD (Figure 3). Most respondents reported prescribing a standard dose of intravenous iron: 1000 mg iron in 87.5% of cases. A third of respondents specified prescribing ferric carboxymaltose, 3.6% specified prescribing 1000 mg of either ferric carboxymaltose or ferric derisomaltose, and the rest did not specify the formulation. For oral iron therapy, 74.6% of respondents prescribed a standard dose, but the standard dose varied from 100–200 mg once or thrice daily to 200 mg every other day, usually prescribed as ferrous fumarate. Treatment did not differ between physicians, nurse practitioners, or the type of hospital.

Lastly, 78.8% of respondents reported not measuring phosphate before or after intravenous iron therapy (Supplementary Figure S1). A noteworthy difference was observed between respondents from academic and non-academic hospitals regarding knowledge of the guidelines and hypophosphatemia. More respondents from non-academic hospitals (92.7%) than academic hospitals (56.0%) do not measure phosphate levels. In addition, 25% of respondents from non-academic hospitals were unaware of the ECCO guidelines compared with 4% of respondents from academic hospitals. However, these differences were not statistically significant after correction for multiple testing.

## 4. Discussion

Six years after the publication of ECCO guidelines on anemia management, we evaluated the prevalence of ID and anemia in outpatients with IBD as well as routine ID(A) care across 16 Dutch hospitals. One in five outpatients with IBD had anemia, but medical professionals did not always characterize it. Iron parameters were measured in 59% of cases: almost half of the patients had absolute ID—the primary cause of anemia in this population—however, a quarter of medical professionals reported not treating it. In contrast, most medical professionals treated (a)symptomatic anemia regardless of severity. A common practice for treating ID(A) was a standard 1000 mg dose of intravenous iron without monitoring for post-treatment hypophosphatemia. Contrary to the ECCO guidelines, oral iron therapy was frequently prescribed in biochemically active IBD and often in quantities exceeding 100 mg of daily elemental iron. We observed a noteworthy difference regarding a relative lack of knowledge of the guidelines and hypophosphatemia by medical professionals working at non-academic hospitals compared with academic hospitals. Collectively, these data demonstrate that raising awareness about ID(A) diagnosis and management is necessary to optimize and personalize routine care.

In this study, anemia was prevalent in 18% of outpatients with IBD. Our results are in line with previous studies, which showed prevalence between 11.7% and 24.6% [7,22,23,24,25,26,27,28,29]. However, higher anemia prevalence (30–65%) was reported in hospitalized and recently diagnosed patients or studies performed exclusively in tertiary care centers [30,31,32,33]. In comparison, lower anemia prevalence has been observed in outpatients at primary care or referral centers [23]. Our study focused on outpatients with established IBD and included patients from various care centers, which might explain why the results are on the lower end of the spectrum. In addition, we found no clear differences in ID(A) prevalence between IBD subtypes except for higher anemia prevalence in stricturing CD compared with inflammatory CD. Several studies also found higher anemia prevalence in patients with stricturing and penetrating CD or E2–E3 ulcerative colitis [22,26,32,34]. Several previous studies have reported a higher prevalence of anemia in patients with CD, but we found no difference between patients with CD and UC. [24,25,26,27,31,32,33,35,36]. Even though median CRP was slightly higher in patients with CD than UC, FCP levels did not differ between patients, which might explain the lack of difference in anemia prevalence. On the other hand, we found that males had anemia more frequently compared with females. In this study, we defined anemia based on the Dutch national reference range, which classifies male patients as anemic at a higher hemoglobin value compared with the World Health Organization definition, which was used in other studies [37]. The association between the male gender and anemia should be investigated further as some authors reported higher prevalence in females, while other authors did not [22,25,26,32,36].

Furthermore, we found that ID was highly prevalent (43.4%) among outpatients with IBD. These results are consistent with previous studies, which reported ID prevalence between 26.5% and 62.5% [7,24,25,27,28,29]. The only studies reporting higher ID prevalence (up to 90%) were published before 2006 [1]. The variance in reported ID prevalence could be explained by heterogeneous populations and differences in ID definitions. Our data show that ID is more prevalent among females. Higher ID prevalence in women could be attributed to menstrual blood loss in addition to gastrointestinal bleeding caused by IBD, but it should not be normalized and left untreated since ID(A) has been associated with lower (health-related) QoL [6,7,8,9,10]. In short, despite (inter-)national treatment guidelines, the prevalence of ID in IBD—especially in female patients—has remained high over the past decade.

Biochemical inflammation is a major risk factor for anemia and ID in patients with IBD. Comparing iron-deficient and iron-sufficient patients, we observed statistically significant differences in CRP, WBC, platelets, and FCP. These findings are consistent with previous studies, which showed associations between anemia and elevated CRP, Erythrocyte Sedimentation Rate, or clinical disease scores, such as the Crohn’s Disease Activity Index [25,32,33,36]. In addition, we found that anemia and ID were associated with a twofold increase in FCP and platelets in multivariable analysis. Interestingly, reactive thrombocytosis has been associated with both: (iron-deficiency) anemia and inflammation [38]. Thrombocytosis should not be overlooked in routine IBD care; however, whether elevated platelets could indicate early iron deficiency in patients with IBD should be evaluated further.

Moreover, our results indicate that current ID(A) management is suboptimal. Iron parameters were not measured in approximately half of the cases, suggesting medical professionals do not always characterize anemia. Our data show that (ID)A is usually treated with a standard dose of intravenous iron, which might not always be enough to replenish the total iron deficit. In 2013, Stein et al. looked at routine care for ID(A) in patients with IBD across nine European countries and reported that 76% of gastroenterologists aimed for Hb normalization, but only 23% sought to normalize ferritin concentrations [39]. In contrast to the latest ECCO guidelines suggesting to treat IDA until iron stores are sufficient, the primary treatment goal among medical professionals still seems to be the normalization of Hb rather than iron stores, which is supported by our findings and a low percentage of reported treatment of non-anemic ID.

Finally, intravenous iron therapy, especially ferric carboxymaltose, is associated with post-treatment hypophosphatemia that can cause or exacerbate fatigue or even lead to life-threatening complications such as heart arrhythmias [40]. In this study, a third of respondents reported prescribing ferric carboxymaltose, but phosphate was measured only in 5.8% of patients treated with intravenous iron. We also found that most patients received daily oral iron—sometimes in doses exceeding 100 mg elemental iron—that is associated with poorer fractional iron absorption due to temporary hepcidin increases, which is why intermittent dosing is preferred [41,42]. Monitoring for post-treatment hypophosphatemia is not a part of the ECCO guidelines, given that the prescribing information was updated in 2020; however, scientific evidence suggests the need to include hypophosphatemia management in the updated treatment guidelines [43]. In short, ID(A) diagnosis and treatment should be improved in routine IBD care.

This is the first study evaluating and comparing routine ID(A) management according to the latest ECCO guidelines. In addition, this study included a large number of outpatients from various care centers across the country, which represents the Dutch IBD population and the routine management by medical professionals. However, this study has several limitations. Firstly, the cross-sectional nature of the study meant that we extracted data from a specific time period and could have missed data on iron therapy, e.g., if a patient received treatment just before or after the cross-sectional period, then we might have missed the data and classified patients as untreated. Secondly, not all cases of anemia could be characterized due to missing data (Supplementary Table S1); however, the missing data illustrates issues in routine management. Lastly, we did not extract data on endoscopic or clinical disease activity and IBD therapy, such as use of thiopurines, corticosteroids, or biologicals, as well as a history of blood transfusions, use of erythropoietin-stimulating agents or proton-pump inhibitors. These data could have provided additional information on associations between ID(A), disease activity, and IBD therapy.

Despite the limitations, this study shows that anemia and ID remain highly prevalent in patients with IBD under ambulatory care. The prevalence has not changed considerably over the years, even though (inter-)national guidelines have been established and reviewed several times. The ECCO guidelines advise regular screening and prompt IDA treatment to normalize not only Hb but also iron stores. This study shows that while IDA is treated, the characterization of anemia and awareness of iron status optimization as a treatment goal is lacking among Dutch medical professionals. It emphasizes the need to address the common misconceptions regarding ID(A) treatment [44]. Finally, this study shows that biochemical inflammation is a major risk factor for anemia and ID, but it is seldom taken into consideration when prescribing iron therapy. Hence, raising awareness about the management of ID(A) is necessary to optimize and personalize routine care.

## Figures and Tables

**Table 1:** Study population characteristics.

	IBD (*n* = 2197)	CD (*n* = 1271)	UC (*n* = 926)	*p*-Value	Available Data (*n*)
**Gender: female** (*n*, %)	1272 (57.9%)	780 (61.4%)	492 (53.1%)	***p* < 0.001**	2197
**Age** (years)	44.0 [31.0–57.0]	42.0 [29.0–55.0]	48.0 [33.0–61.0]	***p* < 0.001**	2196
**Age at diagnosis**				***p* < 0.001**	2142
<17 years old	285 (13.3%)	212 (17.1%)	73 (8.1%)		
17–40 years old	1292 (60.3%)	777 (62.7%)	515 (57.0%)		
>40 years old	565 (26.4%)	250 (20.2%)	315 (34.9%)		
**Disease location**					1245
Terminal ileum		464 (37.3%)			
Colon		260 (20.9%)			
Ileocolon		521 (41.8%)			
upper GI-involvement *		112 (9.8%)			1138
**Disease behavior**					1113
Inflammatory		652 (58.6%)			
Stricturing		318 (28.6%)			
Penetrating		143 (12.8%)			
Perianal disease **		284 (24.9%)			1142
**Disease extension**					885
Ulcerative proctitis			123 (13.9%)		
Left-sided colitis			347 (39.2%)		
Pancolitis			415 (46.9%)		
**Anemia** (*n*, %)	393 (18.0%)	236 (18.7%)	157 (17.0%)	**NS**	2186
Females	183 (14.5%)	124 (16.0%)	59 (12.0%)	*p* < 0.05	
Males	210 (22.8%)	112 (22.9%)	98 (22.8%)	NS	
**Iron deficiency** (*n*, %)	566 (43.4%)	333 (43.4%)	233 (43.5%)	**NS**	1303
Females	396 (51.8%)	236 (49.8%)	160 (55.2%)	NS	
Males	170 (31.5%)	97 (33.1%)	73 (29.7%)	NS	
**Iron-deficiency anemia** (*n*, %)	159 (12.2%)	95 (12.4%)	64 (11.9%)	**NS**	1303
Females	90 (11.8%)	59 (12.4%)	31 (10.7%)	NS	
Males	69 (12.8%)	36 (12.3%)	33 (13.4%)	NS	
**Biochemical inflammation** (*n*, %)	848 (39.8%)	526 (42.3%)	322 (36.3%)	***p* < 0.01**	2132
Females	521 (42.1%)	340 (44.7%)	181 (38.0%)	*p* < 0.05	
Males	327 (36.5%)	186 (38.5%)	141 (34.2%)	NS	
Iron deficiency	384 (70.8%)	223 (68.0%)	161 (75.2%)	NS	1303
Anemia	201 (23.7%)	126 (24.0%)	75 (23.3%)	NS	2186

Continuous variables are represented by the median value and an interquartile range [Quartile 1–Quartile 3]. IBD: Inflammatory Bowel Disease, CD: Crohn’s Disease, UC: includes patients with Ulcerative Colitis and IBD-unclassified, GI: gastrointestinal tract. Anemia: hemoglobin < 7.5 mmol/L and <8.5 mmol/L for females and males, respectively. Iron deficiency: primarily defined by ferritin < 30 µg/L (without biochemical inflammation) or ferritin < 100 µg/L (with biochemical inflammation) or, if ferritin is unavailable, by transferrin saturation < 20% regardless of biochemical inflammation. Biochemical inflammation: defined by C-reactive protein > 5 mg/L and/or fecal calprotectin >150 mg/kg, Iron-deficiency anemia: concurrent anemia and iron deficiency. NS: statistically non-significant (*p*-value > 0.05). *p*-values in **bold** highlight statistical significance after adjustment for multiple testing. *: upper GI-involvement (Montreal L4) is presented as a modifier, which includes isolated upper GI-involvement and upper GI-involvement in addition to other disease locations. **: perianal disease (Montreal p) is presented as a modifier—indicating solely perianal disease involvement separately from penetrating disease behavior.

**Table 2:** Differences between patients with Inflammatory Bowel Disease stratified by iron status.

	Iron-Deficient (*n* = 566)	Iron-Sufficient (*n* = 737)	*p*-Value	Missing Data (*n*, %)
Hemoglobin (mmol/L)	8.20 [7.70–8.80]	8.80 [8.20–9.40]	***p* < 0.001**	1 (0.1%)
Females	8.10 [7.50–8.50]	8.30 [7.90–8.80]	***p* < 0.001**	
Males	8.70 [8.00–9.20]	9.30 [8.60–9.70]	***p* < 0.001**	
Hematocrit (%)	0.41 [0.38–0.43]	0.43 [0.40–0.46]	***p* < 0.001**	129 (9.9%)
MCV (fL)	90.00 [86.00–93.00]	92.00 [89.00–96.00]	***p* < 0.001**	6 (0.5%)
WBC (×10^9^/L)	7.50 [6.30–9.40]	6.80 [5.60–8.30]	***p* < 0.001**	18 (1.4%)
Platelets (×10^9^/L)	310.00 [262.00–362.00]	267.00 [226.00–312.00]	***p* < 0.001**	19 (1.5%)
LDH (U/L)	187.00 [156.00–205.50]	177.00 [149.50–199.00]	NS	1089 (83.6%)
CRP (mg/L)	3.80 [1.00–8.00]	2.00 [1.00–4.00]	***p* < 0.001**	20 (1.5%)
FCP (mg/kg)	291.00 [84.00–924.25]	55.00 [22.00–156.25]	***p* < 0.001**	573 (44.0%)
Ferritin (µg/L)	27.00 [16.00–52.50]	112.00 [66.00–178.00]	***p* < 0.001**	29 (2.2%)
Tsat (%)	17.00 [11.00–23.75]	27.00 [20.00–35.00]	***p* < 0.001**	891 (68.4%)
Iron (µmol/L)	11.25 [7.00–15.98]	16.70 [13.00–21.35]	***p* < 0.001**	830 (63.7%)
Transferrin (g/L)	2.80 [2.50–3.10]	2.40 [2.20–2.70]	***p* < 0.001**	930 (71.4%)
TIBC (µmol/L)	72.20 (± 11.27)	62.00 [57.00–68.00]	***p* < 0.001**	1088 (83.5%)
Folic acid (nmol/L)	13.00 [8.90–21.55]	15.20 [11.15–23.13]	NS	1011 (77.6%)
Vitamin B12 (pmol/L)	336.00 [240.00–483.00]	344.00 [252.00–468.00]	NS	753 (57.8%)

Continuous variables are represented by the mean value and (±Standard Deviation, SD) or by the median value and an interquartile range [Quartile 1–Quartile 3], based on the normality of its distribution. MCV: Mean Corpuscular Volume, CRP: C-reactive Protein, WBC: white blood cell count, Tsat: transferrin saturation, FCP: fecal calprotectin. NS: statistically non-significant (*p*-value > 0.05). *p*-values in **bold** highlight statistical significance after adjustment for multiple testing.

**Table 3:** Risk factors for anemia, iron deficiency, and iron-deficiency anemia in patients with Inflammatory Bowel Disease.

	Anemia			
	Univariable OR (95% CI)	*p*-Value	Multivariable OR (95% CI)	*p*-Value
Gender (male reference)	0.57 [0.46–0.71]	*p* < 0.001	0.43 [0.28–0.66]	*p* < 0.001
Age (years)	1.01 [1.00–1.02]	*p* < 0.01	1.02 [1.01–1.04]	*p* < 0.001
MCV (fL)	0.94 [0.92–0.96]	*p* < 0.001	0.94 [0.91–0.97]	*p* < 0.001
Log_2_ WBC (×10^9^/L)	1.01 [0.80–1.28]	NS		
Log_2_ Platelets (×10^9^/L)	2.12 [1.61–2.78]	*p* < 0.001	2.17 [1.25–3.79]	*p* < 0.01
Log_2_ Ferritin (µg/L)	0.74 [0.68–0.81]	*p* < 0.001	0.75 [0.65–0.87]	*p* < 0.001
Tsat (%)	0.92 [0.90–0.94]	*p* < 0.001		
Log_2_ CRP (mg/L)	1.23 [1.15–1.32]	*p* < 0.001		
Log_2_ FCP (mg/kg)	1.23 [1.15–1.32]	*p* < 0.001	1.18 [1.08–1.28]	*p* < 0.001
	Iron Deficiency			
	Univariable OR (95% CI)	*p*-Value	Multivariable OR (95% CI)	*p*-Value
Gender (male reference)	2.34 [1.86–2.94]	*p* < 0.001	2.63 [1.84–3.75]	*p* < 0.001
Age (years)	0.97 [0.97–0.98]	*p* < 0.001	0.98 [0.97–0.99]	*p* < 0.001
MCV (fL)	0.92 [0.90–0.93]	*p* < 0.001	0.94 [0.91–0.97]	*p* < 0.001
Log_2_ WBC (×10^9^/L)	2.13 [1.67–2.72]	*p* < 0.001		
Log_2_ Platelets (×10^9^/L)	3.79 [2.80–5.14]	*p* < 0.001	1.85 [1.16–2.95]	*p* < 0.01
Log_2_ CRP (mg/L)	1.31 [1.22–1.41]	*p* < 0.001		
Log_2_ FCP (mg/kg)	1.39 [1.30–1.49]	*p* < 0.001	1.39 [1.29–1.50]	*p* < 0.001
	Iron-Deficiency Anemia			
	Univariable OR (95% CI)	*p*-Value	Multivariable OR (95% CI)	*p*-Value
Gender (male reference)	0.91 [0.65–1.27]	NS		
Age (years)	1.00 [0.99–1.01]	NS		
MCV (fL)	0.85 [0.83–0.88]	*p* < 0.001	0.87 [0.84–0.91]	*p* < 0.001
Log_2_ WBC (×10^9^/L)	1.49 [1.04–2.12]	*p* < 0.05		
Log_2_ Platelets (×10^9^/L)	3.52 [2.30–5.40]	*p* < 0.001		
Log_2_ CRP (mg/L)	1.26 [1.15–1.39]	*p* < 0.001		
Log_2_ FCP (mg/kg)	1.37 [1.25–1.51]	*p* < 0.001	1.35 [1.22–1.49]	*p* < 0.001

Odds ratios (OR) are presented with a 95% confidence interval (95% CI). IBD: Inflammatory Bowel Disease, MCV: Mean Corpuscular Volume, CRP: C-reactive Protein, WBC: white blood cell count, Tsat: transferrin saturation, FCP: fecal calprotectin. NS: statistically non-significant (*p*-value > 0.05). The OR for variables expressed in log_2_-scale represents an increase or decrease in the risk of the outcome when the value of a variable doubles.

**Table 4:** Differences between patients with Inflammatory Bowel Disease who received iron therapy and those who did not.

	Received Iron Therapy (*n* = 204)	No Iron Therapy (*n* = 1778)	*p*-Value	Available Data (*n*, %)
Gender: female (*n*, %)	125 (61.3%)	1025 (57.6%)	NS	1982 (100.0%)
Biochemical inflammation (*n*, %)	114 (59.1%)	657 (38.3%)	***p* < 0.001**	1908 (96.3%)
Anemia (*n*, %)	108 (54.3%)	254 (14.5%)	***p* < 0.001**	1949 (98.3%)
Iron-deficiency anemia (*n*, %)	71 (46.7%)	53 (6.9%)	***p* < 0.001**	915 (46.2%)
Iron deficiency (*n*, %)	120 (78.4%)	280 (36.7%)	***p* < 0.001**	916 (46.2%)
Functional iron deficiency (*n*, %)	8 (22.2%)	14 (14.3%)	NS	134 (6.8%)
Folic acid deficiency (*n*, %)	1 (2.0%)	2 (0.9%)	NS	263 (13.3%)
Vitamin B12 deficiency (*n*, %)	5 (7.6%)	22 (6.0%)	NS	433 (21.8%)
Oral iron (*n*, %)	90 (44.1%)	NA	NA	204 (100.0%)
Intravenous iron (*n*, %)	104 (51.0%)	NA	NA	204 (100.0%)
Combined therapy (*n*, %)	10 (4.9%)	NA	NA	204 (100.0%)
Phosphate measurement * (*n*, %)	6 (5.8%)	NA	NA	114 (100.0%)

NA: not applicable, NS: non-significant. Biochemical inflammation: defined by C-reactive protein >5 mg/L and/or fecal calprotectin >150 mg/kg; anemia: defined by hemoglobin <7.5 mmol/L or <8.5 mmol/L for females and males, respectively; iron deficiency: defined primarily by ferritin <30 µg/L (without inflammation) or <100 µg/L (with inflammation) or, if ferritin is unavailable, transferrin saturation <20% regardless of biochemical inflammation. Iron-deficiency anemia: concurrent anemia and iron deficiency. Functional iron deficiency: defined as ferritin >100µg/L with transferrin saturation <20% in the presence of biochemical inflammation. Folic acid deficiency: folic acid level < 5 nmol/L; vitamin B12 deficiency: vitamin B12 level <150 pmol/L. Combined therapy: treatment with oral and intravenous iron. *p*-values in **bold** highlight statistical significance after adjustment for multiple testing. *: represents the number of patients who had their phosphate levels measured after therapy with intravenous iron.

## Data Availability

The datasets analyzed in this study are available from the corresponding author upon reasonable request.

## Supplementary Materials

The following supporting information can be downloaded at: https://www.mdpi.com/article/10.3390/jcm11226843/s1. All supplementary tables as referred to in the manuscript (Supplementary Tables S1–S5) can be found in the separately uploaded document entitled: ‘Supplementary Data Content 1’. All supplementary figures as referred to in the manuscript (Supplementary Figure S1) can be found in the separately uploaded document entitled: ‘Supplementary Data Content 1’.
