# Supplementary material for: Anemia and Iron Deficiency in Outpatients with Inflammatory Bowel Disease: Ubiquitous Yet Suboptimally Managed
Source: J Clin Med. 2022 Nov 19;11(22):6843. doi: 10.3390/jcm11226843 (PMC9692778; doi:10.3390/jcm11226843)
Supplement: Supplementary file 1 [file jcm-11-06843-s001.zip › Supplementary Data Content 1.pdf]

SUPPLEMENTARY TABLES

Supplementary Table S1. Missing data

Variable (*n*, %)	Missing data IBD (*n* = 2197)	Missing data CD (*n* = 1271)	Missing data UC (*n* = 926)
Gender	0 (0.0%)	0 (0.0%)	0 (0.0%)
Age	1 (0.05%)	1 (0.08%)	0 (0.0%)
Montreal A	55 (2.5%)		
Montreal L		26 (2.0%)	
Montreal L4		133 (10.5%)	
Montreal B		158 (12.4%)	
Montreal p		129 (10.1%)	
Montreal E			41 (4.4%)
**Biochemical data (at inclusion)**			
Hemoglobin	11 (0.5%)	6 (0.5%)	5 (0.5%)
Hematocrit	317 (14.4%)	201 (15.8%)	116 (12.5%)
MCV	36 (1.6%)	21 (1.7%)	15 (1.6%)
CRP	106 (4.8%)	50 (3.9%)	56 (6.0%)
WBC	56 (2.5%)	27 (2.1%)	29 (3.1%)
Platelets	60 (2.7%)	27 (2.1%)	33 (3.6%)
LDH	1791 (81.5%)	1033 (81.3%)	758 (81.9%)
Ferritin	885 (40.3%)	510 (40.1%)	375 (40.5%)
Iron	1706 (77.7%)	990 (77.9%)	716 (77.3%)
Transferrin	1809 (82.3%)	1057 (83.2%)	752 (81.2%)
TIBC	1980 (90.1%)	1147 (90.2%)	833 (90.0%)
Tsat	1771 (80.6%)	1031 (71.1%)	740 (79.9%)
Folic acid	1866 (84.9%)	1029 (81.0%)	837 (90.4%)
Vitamin B12	1602 (72.9%)	838 (65.9%)	764 (82.5%)
FCP	1074 (48.9%)	623 (49.0%)	451 (48.7%)
Reticulocyte (percentage)	2196 (99.95%)	1271 (100.0%)	925 (99.9%)
Reticulocyte concentration	2181 (99.3%)	1260 (99.1%)	921 (99.5%)
**Data (prior appointment)**	**IBD (*n* = 1995)**	**CD (*n* = 1165)**	**UC (*n* = 830)**
Iron therapy*	13 (0.7%)*	7 (0.6%)*	6 (0.7%)*
Hemoglobin	33 (1.7%)	17 (1.5%)	16 (1.9%)
Hematocrit	297 (14.9%)	184 (15.8%)	113 (13.6%)
MCV	64 (3.2%)	39 (3.4%)	25 (3.0%)
CRP	137 (6.9%)	62 (5.3%)	75 (9.0%)
WBC	170 (8.2%)	82 (7.0%)	88 (10.6%)
Platelets	177 (8.9%)	89 (7.6%)	88 (10.6%)
LDH	1675 (84.0%)	975 (83.7%)	700 (84.3%)
Ferritin	1061 (53.2%)	601 (51.6%)	460 (55.4%)
Iron	1634 (81.9%)	940 (80.7%)	694 (83.6%)
Transferrin	1700 (85.2%)	989 (84.9%)	711 (85.7%)
TIBC	1854 (92.9%)	1072 (92.0%)	782 (94.2%)
Tsat	1672 (83.8%)	967 (83.0%)	705 (84.9%)
Folic acid	1729 (86.7%)	982 (84.3%)	747 (90.0%)
Vitamin B12	1558 (78.1%)	851 (73.0%)	707 (85.2%)
FCP	995 (49.9%)	580 (49.8%)	415 (50.0%)
Reticulocyte (percentage)	1995 (100.0%)	1165 (100.0%)	830 (100.0%)
Reticulocyte concentration	1978 (99.1%)	1155 (99.1%)	823 (99.2%)

IBD: Inflammatory Bowel Disease, CD: Crohn's disease, UC: includes patients with ulcerative colitis and IBD-unclassified. Montreal A: age at diagnosis, Montreal L: disease location, Montreal L4: upper gastrointestinal tract-involvement (modifier), Montreal B: disease behavior, Montreal p: perianal disease (modifier), Montreal E: disease extension. MCV: Mean Corpuscular Volume, CRP: C-reactive protein, WBC: white blood cell count, LDH: lactate dehydrogenase, TIBC: total-iron binding capacity, Tsat: transferrin saturation, FCP: fecal calprotectin. *: includes missing data and iron therapy which has been prescribed during visit 1 instead of visit 2.

Supplementary Table S2. Missing questionnaire responses.

Questionnaire question	Missing data (*n*, %)
Profession	0 (0.0%)
Place of employment	0 (0.0%)
Amount of weekly outpatient IBD visits	0 (0.0%)
Routine screening in quiescent IBD	0 (0.0%)
Frequency routine screening in quiescent IBD	0 (0.0%)
Routine screening in active IBD	0 (0.0%)
Frequency routine screening in active IBD	0 (0.0%)
Awareness and familiarity with the European guidelines	0 (0.0%)
Biochemical parameters for anemia characterization	0 (0.0%)
Treatment of asymptomatic anemia	1 (1.4%)
Treatment of symptomatic anemia	1 (1.4%)
Anemia treatment in quiescent IBD	1 (1.4%)
Anemia treatment in active IBD	1 (1.4%)
Treatment of iron deficiency without anemia	1 (1.4%)
Type of prescribed treatment for iron deficiency without anemia	1 (1.4%)
Standard oral iron therapy regimen	2 (2.9%)
Standard oral iron therapy dose	0 (0.0%)
Standard intravenous iron therapy regimen	2 (2.9%)
Standard intravenous iron therapy dose	0 (0.0%)
Basis for the intravenous iron dose	0 (0.0%)
Phosphate measurements	3 (4.3%)

Supplementary Table S3. Baseline biochemical profile for the study population.

	**IBD** (*n* = 2197)	**CD** (*n* = 1271)	**UC** (*n* = 926)	*P*-value
**Hemoglobin** (mmol/L)	**8.50 [8.00–9.10]**	**8.50 [7.90–9.10]**	**8.60 [8.10–9.20]**	***P* < 0.001**
Females	*8.20 [7.70–8.60]*	*8.20 [7.70–8.60]*	*8.30 [7.80–8.60]*	NS
Males	*9.10 [8.50–9.60]*	*9.10 [8.50–9.50]*	*9.20 [8.58–9.60]*	NS
**Hematocrit** (%)	0.42 [0.39–0.44]	0.42 [0.39–0.44]	0.42 [0.40–0.45]	***P* < 0.01**
**MCV** (fL)	91.00 [88.00–95.00]	91.00 [87.00–95.00]	92.00 [88.00–95.00]	***P* < 0.05**
**WBC** ($\times 10^9$/L)	7.10 [5.70–8.70]	7.20 [5.80–8.80]	6.80 [5.60–8.60]	***P* < 0.001**
**Platelets** ($\times 10^9$/L)	281.00 [234.00–329.00]	284.50 [236.00–334.75]	276.00 [232.00–324.00]	***P* < 0.01**
**LDH** (U/L)	173.00 [151.75–198.00]	169.00 [147.00–194.00]	184.00 [159.00–207.00]	***P* < 0.001**
**CRP** (mg/L)	2.00 [1.00–6.00]	3.00 [1.00–6.20]	2.00 [1.00–4.00]	***P* < 0.001**
**FCP** (mg/kg)	111.00 [32.00–492.00]	111.50 [33.00–435.00]	110.00 [29.00–588.00]	NS
**Ferritin** (µg/L)	62.50 [29.00–122.00]	64.00 [31.00–124.50]	60.00 [27.00–117.00]	NS
**Iron** (µmol/L)	14.40 [10.30–20.00]	13.60 [10.00–19.00]	15.30 [10.90–21.00]	***P* < 0.05**
**Tsat** (%)	22.00 [15.95–31.00]	21.00 [15.00–28.00]	24.50 [17.08–34.00]	***P* < 0.01**
**Transferrin** (g/L)	2.6 [2.30–2.90]	2.60 [2.30–2.90]	2.52 [2.30–2.80]	NS
**TIBC** (µmol/L)	65.00 [58.00–73.00]	66.50 [59.00–74.00]	65.00 [58.00–73.00]	NS
**Folic acid** (nmol/L)	15.00 [10.00–22.50]	14.05 [9.10–23.13]	15.80 [12.40–21.70]	NS
**Vitamin B12** (pmol/L)	337.00 [243.00–477.00]	329.00 [239.50–484.00]	359.50 [248.00–462.75]	NS

IBD: Inflammatory Bowel Disease, CD: Crohn's disease, UC: includes patients with ulcerative colitis and IBD-unclassified. Continuous variables are represented by the mean value and (± Standard Deviation, SD) or by the median value and an interquartile range [Quartile 1– Quartile 3], based on the normality of its distribution. MCV: Mean Corpuscular Volume, CRP: C-reactive Protein, WBC: white blood cell count, LDH: lactate dehydrogenase, Tsat: transferrin saturation, FCP: fecal calprotectin. Active IBD: biochemically active inflammatory bowel disease, quiescent IBD: biochemically quiescent inflammatory bowel disease. NS: statistically non-significant (*P*-value > 0.05). *P*-values in **bold** highlight statistical significance after adjusting for multiple testing.

Supplementary Table S4. Differences between patients with Inflammatory Bowel Disease, stratified by the presence or absence of anemia.

	**Anemia (*n* = 393)**	**No anemia (*n* = 1793)**	***P*-value**	**Available data (*n*, %)**
**Age** (years)	46.00 [33.00–61.00]	43.00 [30.00–57.00]	***P* < 0.01**	2185 (99.95%)
**Gender: female** (*n*, %)	183 (46.6%)	1083 (60.4%)	***P* < 0.01**	2186 (100.0%)
**Biochemical inflammation** (*n*, %)	201 (53.3%)	646 (37.0%)	***P* < 0.001**	2124 (97.2%)
**MCV** (fL)	89.00 [84.00–94.00]	91.00 [88.00–95.00]	***P* < 0.001**	2161 (98.9%)
**WBC** ($\times 10^9$/L)	7.16 [5.60–9.00]	7.01 [5.70–8.60]	NS	2141 (97.9%)
**Platelets** ($\times 10^9$/L)	300.50 [247.00–376.25]	277.00 [232.00–324.00]	***P* < 0.001**	2137 (97.8%)
**Ferritin** (µg/L)	37.50 [13.40–106.15]	67.00 [35.00–125.00]	***P* < 0.001**	1309 (59.9%)
**Tsat** (%)	16.00 [7.00–22.08]	25.00 [18.00–33.20]	***P* < 0.001**	425 (19.4%)
**Folic acid** (nmol/L)	14.40 [8.76–20.00]	15.00 [10.35–23.60]	NS	330 (15.1%)
**Vitamin B12** (pmol/L)	318.00 [204.50–473.00]	339.00 [251.50–477.50]	NS	594 (27.2%)
**CRP** (mg/L)	3.00 [1.00–8.98]	2.00 [1.00–5.00]	***P* < 0.001**	2091 (95.7%)
**FCP** (mg/kg)	312.50 [73.75–1144.75]	93.00 [28.50–409.00]	***P* < 0.001**	1115 (51.1%)

Continuous variables are represented by the mean value and (± Standard Deviation, SD) or by the median value and an interquartile range [Quartile 1– Quartile 3], based on the normality of its distribution. Biochemical inflammation: defined as C-reactive protein > 5mg/L and/or fecal calprotectin >150mg/kg. MCV: Mean Corpuscular Volume, CRP: C-reactive Protein, WBC: white blood cell count, Tsat: transferrin saturation, FCP: fecal calprotectin. NS: statistically non-significant (*P*-value > 0.05). *P*-values in **bold** highlight statistical significance after adjusting for multiple testing.

Supplementary Table S5. Montreal classification and the risk of anemia, iron deficiency, and iron-deficiency anemia in patients with Inflammatory Bowel Disease.

	**Anemia**		**Iron deficiency**		**Iron-deficiency anemia**	
	Univariable OR (95% CI)	*P*-value	Univariable OR (95% CI)	*P*-value	Univariable OR (95% CI)	*P*-value
**Age at diagnosis**						
< 17 years old	Reference		Reference		Reference	
17–40 years old	1.05 [0.75–1.47]	NS	0.74 [0.52–1.04]	NS	1.03 [0.61–1.73]	NS
> 40 years old	1.11 [0.76–1.61]	NS	0.48 [0.33–0.71]	*P* < 0.001	0.79 [0.43–1.43]	NS
**Disease location**						
Terminal ileum	Reference		Reference		Reference	
Colon	0.75 [0.50–1.11]	NS	0.85 [0.57–1.26]	NS	0.70 [0.39–1.28]	NS
Ileocolon	0.83 [0.60–1.14]	NS	1.18 [0.85–1.62]	NS	0.78 [0.48–1.25]	NS
**Upper GI-involvement***	1.08 [0.67–1.76]	NS	1.24 [0.75–2.03]	NS	1.46 [0.75–2.84]	NS
**Disease behavior**						
Inflammatory	Reference		Reference		Reference	
Stricturing	1.55 [1.12–2.16]	*P* < 0.01	1.17 [0.83–1.64]	NS	1.37 [0.84–2.24]	NS
Penetrating	1.28 [0.81–2.02]	NS	0.70 [0.43–1.16]	NS	0.83 [0.38–1.82]	NS
**Perianal disease****	0.93 [0.66–1.31]	NS	0.93 [0.66–1.31]	NS	0.67 [0.38–1.17]	NS
**Disease extension**						
Ulcerative Proctitis	Reference		Reference		Reference	
Left-sided colitis	0.77 [0.44–1.35]	NS	0.83 [0.48–1.43]	NS	0.84 [0.35–2.00]	NS
Pancolitis	1.19 [0.70–2.01]	NS	1.00 [0.59–1.70]	NS	1.35 [0.60–3.06]	NS

The Odds Ratios (OR) are presented with 95% confidence interval (95% CI). NS: statistically non-significant (*P*-values > 0.05). *: upper GI involvement (Montreal L4) is presented as a modifier, which includes isolated upper GI-involvement or upper GI-involvement in addition to other disease locations. **: perianal disease (Montreal p) is presented as a modifier—indicating solely perianal disease involvement separately from penetrating disease behavior.

SUPPLEMENTARY FIGURES

Supplementary figure S1. Respondent data regarding iron-deficiency anemia management in patients with Inflammatory Bowel Disease.

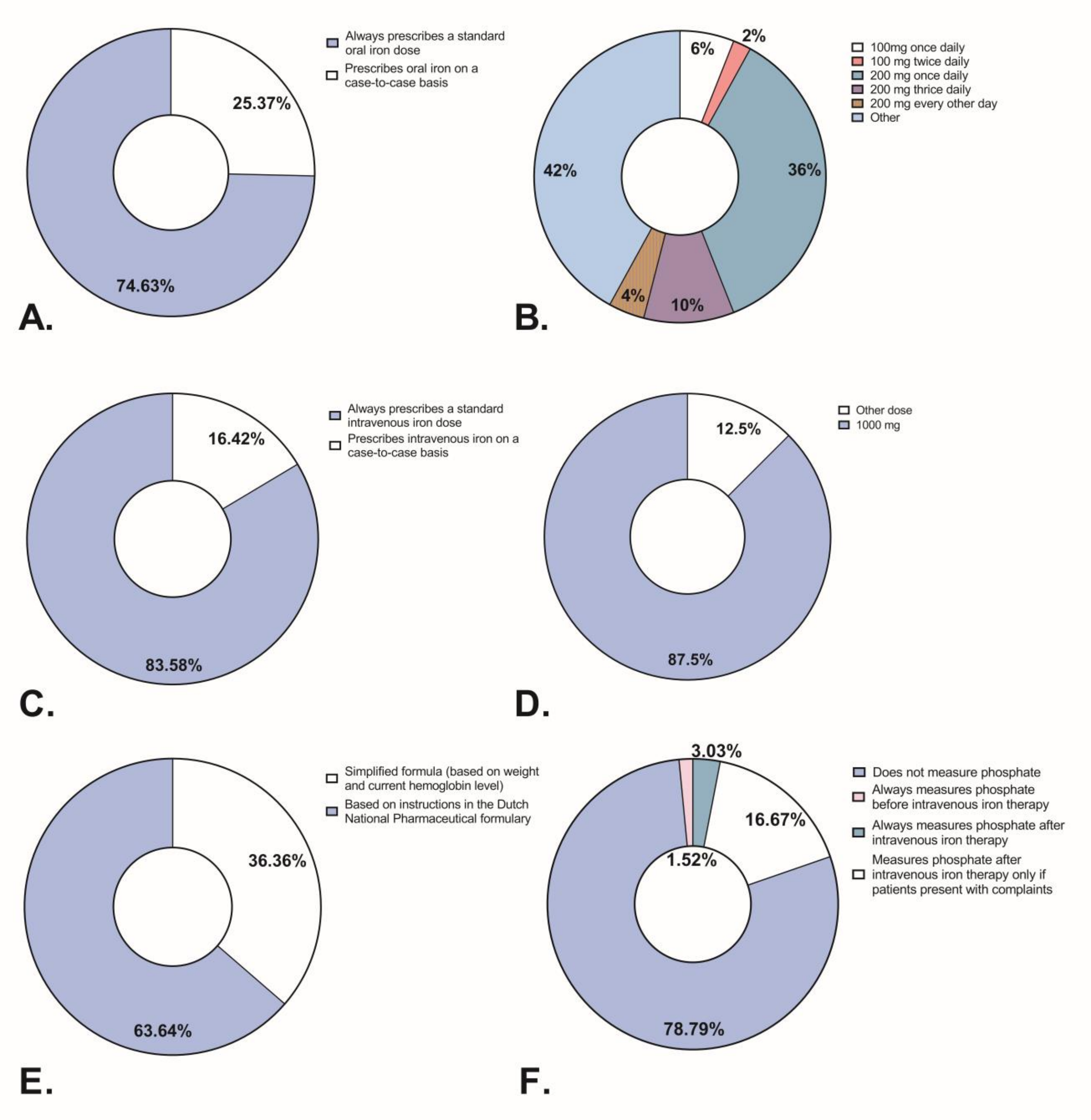

(**A**) Depicts the responses regarding standard or personalized approach to oral iron therapy. (**B**) Depicts the responses regarding the standard dosage of oral iron. (**C**) Depicts the responses regarding standard or personalized approach to intravenous iron therapy. (**D**) Depicts the responses regarding the standard dosage of intravenous iron. (**E**) Depicts the responses regarding calculations used for prescribing intravenous iron therapy. (**F**) Depicts the responses regarding phosphate measurement in the context of intravenous iron therapy.
